# In Silico Study of the *RSH* (*RelA*/*SpoT* Homologs) Gene Family and Expression Analysis in Response to PGPR Bacteria and Salinity in *Brassica napus*

**DOI:** 10.3390/ijms221910666

**Published:** 2021-10-01

**Authors:** Grażyna B. Dąbrowska, Sena Turkan, Wioleta Tylman-Mojżeszek, Agnieszka Mierek-Adamska

**Affiliations:** Department of Genetics, Faculty of Biological and Veterinary Sciences, Nicolaus Copernicus University in Toruń, Lwowska 1, 87-100 Toruń, Poland; browsk@umk.pl (G.B.D.); senaturkan@doktorant.umk.pl (S.T.); tylman@doktorant.umk.pl (W.T.-M.)

**Keywords:** rapeseed, RelA/SpoT homolog, RSH, alarmones, salinity, stringent response, PGPR

## Abstract

Among several mechanisms involved in the plant stress response, synthesis of guanosine tetra and pentaphosphates (alarmones), homologous to the bacterial stringent response, is of crucial importance. Plant alarmones affect, among others, photosynthetic activity, metabolite accumulation, and nutrient remobilization, and thus regulate plant growth and development. The plant *RSH* (*RelA*/*SpoT* homolog) genes, that encode synthetases and/or hydrolases of alarmones, have been characterized in a limited number of plant species, e.g., *Arabidopsis thaliana*, *Oryza sativa,* and *Ipomoea nil*. Here, we used dry-to-wet laboratory research approaches to characterize *RSH* family genes in the polyploid plant *Brassica napus*. There are 12 *RSH* genes in the genome of rapeseed that belong to four types of *RSH* genes: 6 *RSH1*, 2 *RSH2*, 3 *RSH3*, and 1 *CRSH*. *BnRSH* genes contain 13–24 introns in *RSH1*, 2–6 introns in *RSH2*, 1–6 introns in *RSH3*, and 2–3 introns in the *CRSH* genes. In the promoter regions of the *RSH* genes, we showed the presence of regulatory elements of the response to light, plant hormones, plant development, and abiotic and biotic stresses. The wet-lab analysis showed that expression of *BnRSH* genes is generally not significantly affected by salt stress, but that the presence of PGPR bacteria, mostly of *Serratia* sp., increased the expression of *BnRSH* significantly. The obtained results show that *BnRSH* genes are differently affected by biotic and abiotic factors, which indicates their different functions in plants.

## 1. Introduction

Several species belonging to the *Brassicaceae* Burnett family are economically important plants, i.e., oil and fodder plants in agriculture, vegetables in horticulture including herbal species, and plants used in floriculture. The model plant *A. thaliana* also belongs to this plant family. The genus *Brassica* contains 37 species; the most extensively cultivated are *B. rapa* L., *B. juncea* L. Czernj & Cosson (mustard plant), *B. napus* L. (oilseed rape, rape, rapeseed, canola), and *B. carinata* A. Braun (Abyssinian cabbage) [1]. Rapeseed is a crop plant cultivated in temperate and subtropical regions, mainly for oil production purposes, as seeds of this plant are rich in fat (40–49%). The rapeseed oil is used in both the food industry, as it is one of the healthiest oils, and the energy industry, to produce biofuel. Rape oil by-products are utilised for the production of fodder due to their high protein content [2]. Rapeseed is cultivated all over the world, depending on climatic conditions and latitudes; three types, i.e., the winter, semi-winter, and spring types, are cultivated with varying intensity [3]. *B. napus* is an allopolyploid plant (A_r_A_r_C_o_C_o_); its genome is a result of *B. oleracea* (Mediterranean cabbage, C_o_C_o_) and *B. rapa* (A_r_A_r_) genome hybridization, followed by duplication. The genome of *B. napus* has already been sequenced [4].

The crop yield depends strictly on the ability of plants to adapt to adverse and changeable environmental conditions, which is especially important during seed germination, and during the first stages of plant growth and development. Soil salinity is one of the crucial environmental stresses that have severely decreased crop productivity all over the world. It negatively affects plant physiology and metabolism, including photosynthesis, lipid metabolism, protein synthesis, and nitrogen fixation [5]. The abundance of Na^+^ and Cl^−^ inhibits absorption of other macronutrients causing nutritional imbalance. Moreover, salinity leads to water stress, increased reactive oxygen species production, and oxidative stress [6,7].

Plant growth-promoting rhizobacteria (PGPR) exert several beneficial effects on host plants by promoting plant growth and development, including in stress conditions, via varied mechanisms, such as the production of phytohormones, secondary metabolites, and antibiotics [8,9,10]. Plant growth promoting bacteria, especially halotolerant bacteria, could be a crucial factor for improving plant tolerance to salt stress in an environmentally friendly way [8,9]. PGPR isolated from the rice rhizosphere improved the growth of rice plants exposed to salt stress by lowering the level of ethylene [10]. *Serratia liquefaciens* KM4 increased the growth and biomass of maize grown in salt-stress conditions, and the increased expression of plant stress-related genes has been observed [6]. The inoculation of lettuce with *Pseudomonas mendocina* has a greater effect on plant growth in salt stress conditions than inoculation with arbuscular mycorrhizal fungi. In the presence of analysed PGPR the induction of a plant antioxidant system was observed, even in severe salinity conditions [11]. The inoculation of tomato with PGPR, especially *Arthobacter* sp. and *Pseudomonas* sp., under salinity stress outperformed chemical fertilization [12].

Organisms living in a fluctuating environment have evolved a range of mechanisms to respond to various stress conditions. Among several other mechanisms in bacteria, one of the most important is the stringent response. It was first described in *Escherichia coli* in response to the absence of amino acids [13]. The response is based on the synthesis of the atypical signalling nucleotides, guanosine tetraphosphates (ppGpp) and guanosine pentaphosphates (pppGpp), called alarmones. The increased amount of alarmones in response to stress conditions leads to the immediate arrest of rRNA, tRNA, and ribosomal protein gene expression, followed by the induction of expression of genes encoding proteins involved in adaptation to unfavourable conditions [14,15]. The metabolism of (p)ppGpp in *E. coli* is regulated by RelA and SpoT enzymes encoded by paralogous genes. RelA is a (p)ppGpp synthetase, whereas SpoT is mainly a (p)ppGpp hydrolase, however, in certain conditions it exhibits low activity of alarmone synthetase. Most bacteria possess only one bifunctional Rel enzyme [16,17,18,19].

The presence of (p)ppGpp in photosynthetic *Eucaryota* was first confirmed in the alga *Chlamydomonas reinhardtii*, where the accumulation of alarmones in response to amino acid starvation was observed [20]. Homologs of the bacterial genes *RelA*/*SpoT* called *RSH* (*RelA*/*SpoT* Homologs) were first identified in *A. thaliana* [21] and, in subsequent years, *RSH* genes have been identified in other plant species [22,23,24,25]. RSH proteins have been divided into three groups, i.e., *RSH1*, *RSH2/3*, and *CRSH*, based on their primary structure and domain structure [26]. In *A. thaliana*, there are four genes encoding RSH proteins, namely *RSH1*, *RSH2*, *RSH3*, and *CRSH* (Ca^2+^-activated *RSH*). RSH1 exhibits only (p)ppGpp hydrolytic activity due to the substitution, critical for (p)ppGpp synthase activity, of glycine by serine in the RSD domain. Proteins belonging to the RSH2/3 group (AtRSH2 and AtRSH3) can both synthesize and hydrolase alarmones, whereas CRSH proteins do not possess a functional hydrolytic domain (HD domain) and are (p)ppGpp synthases [26,27,30]. Members of the RSH1 group possess a TGS domain which has been proposed to play a regulatory role in ligand binding [27], and a role in establishing the RSH-ribosome interaction in chloroplasts [28,29,30]. Moreover, RSH1 as the only group of plant RSH proteins that possess the ACT domain [30], recently described as an RNA recognition motif (RRM) domain [28]. CRSH group proteins also contain the EF-hand motif at the C-terminus of the protein. Interestingly, this Ca^2+^-binding motif has not been identified in any bacterial or plant homolog [26,31]. It was confirmed in vitro that, for (p)ppGpp synthase activity, *CRSH* requires Ca^2+^ [32]. The plant stringent response has been implicated in the stress response, flowering, seed development, photosynthesis, plant senescence, and nutrient remobilization [27].

In animals, homologs of bacterial SpoT have been identified (Mesh1) with alarmone hydrolysing activity [33]. However, until quite recently, the existence of (p)ppGpp in metazoa has been questioned. Last year the presence of ppGpp in *Drosophila* and human cells was shown [34], opening a new chapter in the discussion about the origin and functions of alarmones.

In the present study, we attempt to answer the question about the complexity of the plant RSH groups in representatives of the *Brassicaceae* family via the in silico analysis of *RSH* genes and RSH proteins from selected species of this plant family. Inspired by the postulated role of RSH in the plant response to varied abiotic and biotic factors, we also examined *B. napus RSH* gene expression in response to salinity. Moreover, we analysed the expression of *BnRSHs* in the presence of *Serratia liquefaciens*, *S. plymuthica,* and *Massilia timonae*, PGPR bacteria for which the ability to promote the growth of rape has been confirmed. To pinpoint other potential regulators of *RSH* gene expression, we revealed the presence of multiple putative regulatory *cis*-elements in the promoter regions of *BnRSH* genes.

## 2. Results and Discussion

### 2.1. In Silico Analysis of RSH Genes and Proteins in B. napus and Selected Close Relatives from the Brassicaceae Family

Over 20 years ago, *RelA*/*SpoT* homologs (*RSH*) were discovered in plants [21], and the occurrence of the stringent response in plants was also proposed. Subsequently, *RSH* genes have been characterized in other plant species, and it has been shown that the stringent response plays a critical role in the regulation of plant growth and development, and in adaptation to different environmental niches [23,24]. The nature of the evolutionary basis of the stringent response raises questions regarding the complexity of plant *RSH* gene families including their number, and the structure of plant *RSH* proteins in various plant species. The plant *RSH* proteins have been divided into three groups (*RSH1*, *RSH2/3*, and *CRSH*), based mostly on protein primary structure. The members of these three groups of *RSH* proteins vary in their expression patterns and catalytic activities and, therefore, they probably fulfil distinct physiological roles. It seems that the diversification in plant *RSH* genes occurred when plants adapted to terrestrial conditions, and resulted either in the loss or acquisition of some structural and functional features [35,36]. Here, in order to reveal the complexity of the *RSH* gene family, and to further predict relations between sequence and function, we have analysed in silico *RSH* genes and RSH proteins in *B. napus*, and in selected relatives from the *Brassicaceae* family.

#### 2.1.1. Characteristics of Selected *Brassicaceae RSH* Genes

In silico studies are often used as a preliminary means of analysis of plant gene families that enable the capturing of the phylogenetic relationships within a family of genes in one species, as well as between species [36,37,38,39,40]. A total of 45 *RSH* genes that were identified were selected for this study of *Brassicaceae* (*B. napus*, *B. olearacea*, *B. rapa*, *Camelina sativa*, and *Raphanus sativus*) plants are shown in Table 1. *B. napus* is an allotetraploid species and thus, as expected, has more *RSH* orthologous genes (14 in total, including 2 pseudogenes) than *A. thaliana*, where only 4 *RSH* genes have been described [35,41]. Four *RSH* genes are present also in the *B. rapa* genome, whereas in the genome of *B. oleracea* 6 *RSH* genes occur, and in the genome of *R. sativus* 8 *RSH* genes are present, though all these plants are diploids. In the allohexaploid genome of *C. sativa* 12 *RSH* genes are present, however, 3 of them are pseudogenes. In *O. sativa*, one gene in each of the *RSH1*, *RSH2*, and *RSH3* subgroups, and three *CRSH* genes were identified [42]. In *I. nil*, five *RSH* genes were identified, i.e., 1 *RSH1*, 2 *RSH2*, 1 *RSH3*, and 1 *CRSH* [25]. Genes encoding *RSH* were described also in *Capsicum annum* [43], *Nicotiana tabacum* [44], and *Suaeda japonica* [45].

**Figure 1:**
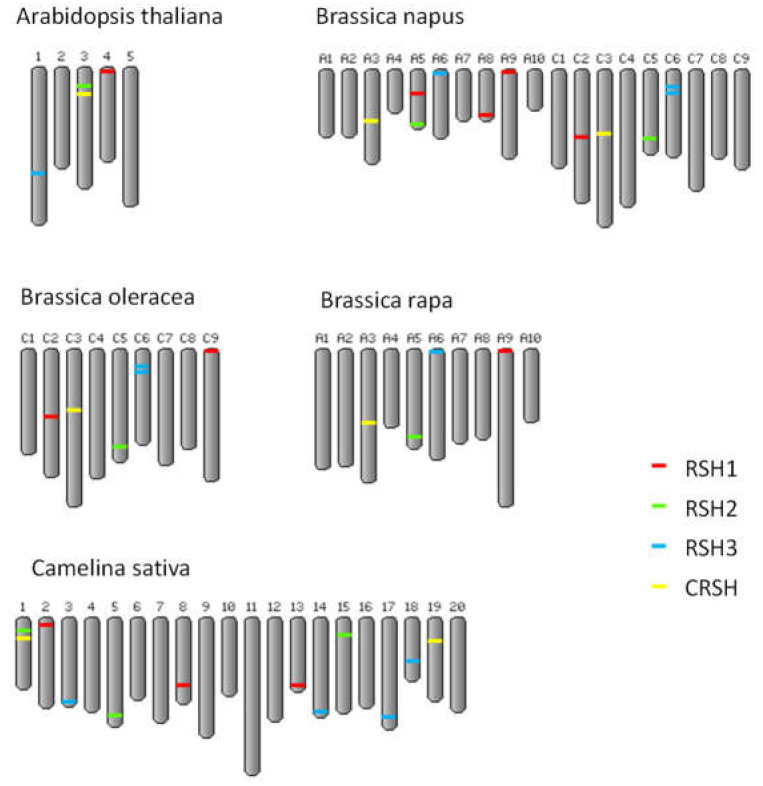
Chromosomal localization of *RSH* genes in *A. thaliana*, *B. napus*, *B. oleracea*, *B. rapa* and *C. sativa*.

*B. napus RSH* genes are distributed in 9 out of 19 chromosomes (Figure 1), but one of the *BnRSH1* genes has not yet been assigned to any chromosome. In *B. oleracea*, *RSH* genes are located on 5 out of 9 chromosomes, and in *B. rapa* the *RSH* genes are located on 4 out of 10 chromosomes. There are no differences between the number and the localization of *RSH* genes on chromosomes in *B. oleracea* and on C-genome chromosomes in *B. napus*. In the case of A-genome chromosomes, there are additional *RSH1* genes on chromosome A5 and A9 in comparison with the genome of. *B. rapa*. Moreover, the *CRSH* gene located on chromosome A3 is a pseudogene in *B. napus*. The presence of an *RSH3* pseudogene located on chromosome A6 could be caused by genome assembly errors since both genes lies in proximity and are separated by an unknown sequence.

**Figure 2:**
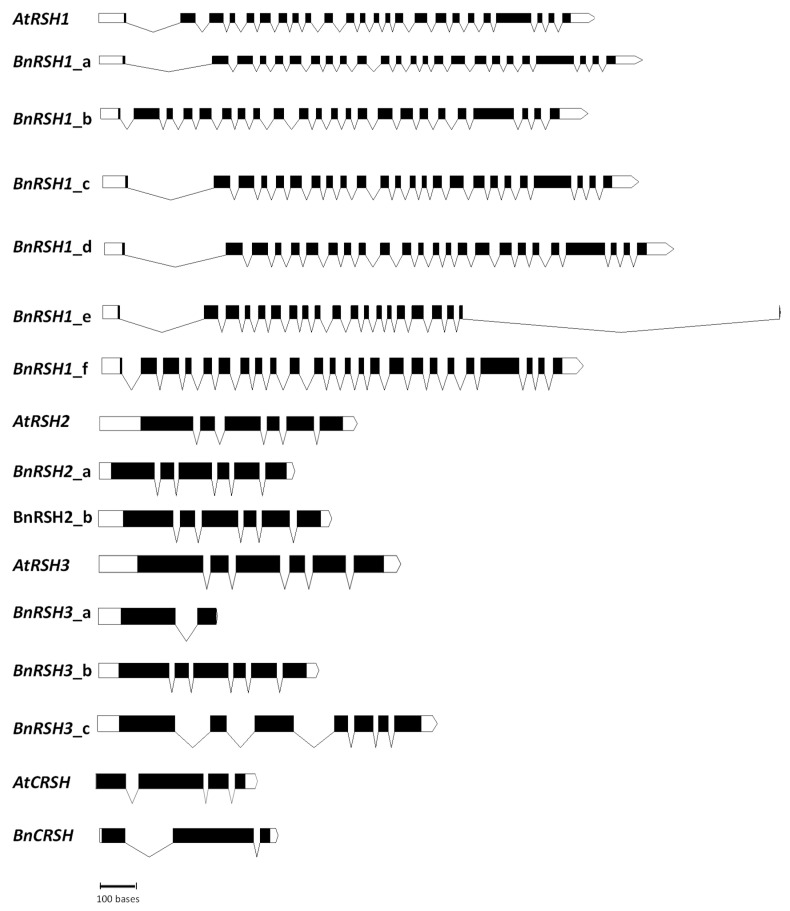
Intron-exon structure of *RSH* genes in *A. thaliana* and *B. napus*. White rectangles indicate UTRs, and black rectangles indicate coding sequence. Intron positions are marked by lines. The analysis was performed using the CIWOG tool.

Further in silico comparative analysis of the intron-exon organization of *RSH* genes in selected *Brassicaceae* species (Figure 2 and Supplementary Figure S1) showed that the number of exons and introns, and the location of introns in different types of *RSH* genes, was preserved in the plants analysed. Plant *RSH1* genes are characterized by very complex structures, with over 20 introns and exons in each analysed gene, except for *BnRSH1_e* and *RsRSH1_c* (Table 1). The high number of introns and exons is a common feature of *RSH1* genes from both mono- and di-cotyledonous plants [25] (data from the NCBI Gene Database). The average number of introns per gene in plants is about 4 [46,47], which raises a question about the possible role of such great complexity in the *RSH1* gene. It is widely accepted that introns fulfil different roles, i.e., introns may contain regulatory elements, they may serve as alternative promoters, or they may be a template for synthesis of non-coding regulatory RNAs [46]. Moreover, introns are crucial for alternative splicing and, in plants, intron retention is a widely observed phenomenon [48]. The presence of introns enhances the expression of genes in varied organisms [49]; however, interestingly, in plants in contrast to animals, higher expression is observed for genes containing more and longer introns [50]. The highly complex structure of *RSH1* genes in plants may suggest their high expression and important roles in many metabolic pathways. Other *RSH* genes in plants are much more compact than *RSH1*, containing approximately 5 introns in *RSH2/3* genes, and 2–3 introns in *CRSH* genes (Table 1, Figure 2 and Supplementary Figure S1).

#### 2.1.2. Characteristic of Selected *Brassicaeae* RSH Proteins

**Figure 3:**
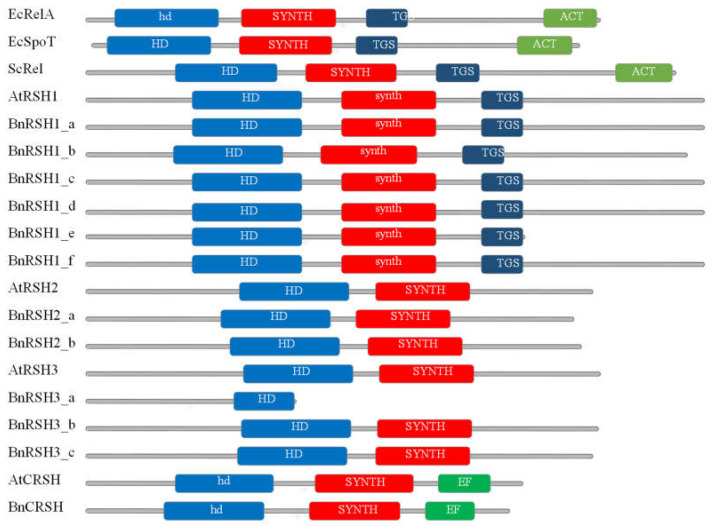
Predicted primary structures of RSH1, RSH2/RSH3, and CRSH proteins from *A. thaliana* and *B. napus*. HD (hd contains HD-SE substitution) (p)ppGpp hydrolase domain; SYNTH (synth contains G-S substitution) (p)ppGpp synthase domain; ACT aspartate kinase chorismate mutase TyrA domain; EF Ca^2+^-binding domain; TGS: threonyl-tRNA synthetase, GTPase, SpoT domain.

In silico studies have shown that all analysed RSH proteins contain the (p)ppGpp hydrolase (HD) and (p)ppGpp synthetase (SYNTH) domains (Figure 3 and Figure S2). RSH1 proteins also possess a TGS domain that is also present in bacterial stringent-response proteins. CRSH proteins contain an EF domain which is specific only for plant CRSH. On the other hand, bacterial RelA and SpoT proteins contain an ACT domain that is not present in any group of plant RSH proteins (Figure 3).

**Figure 4:**
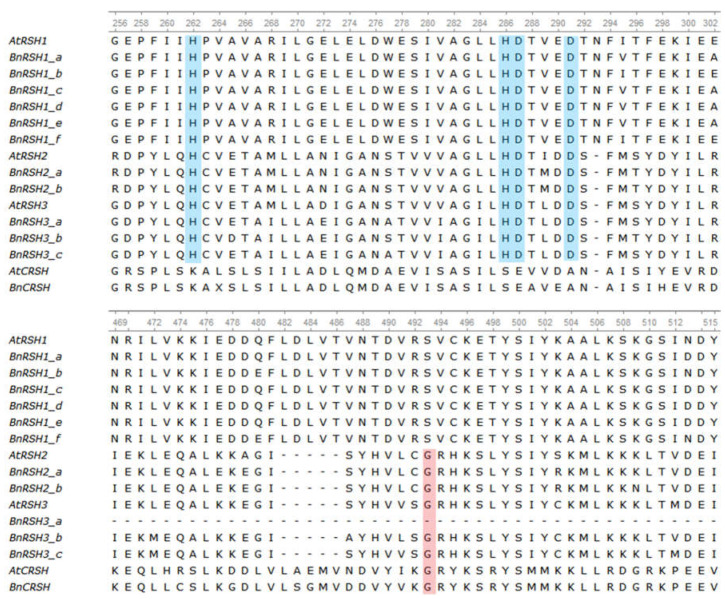
Amino acid alignments for the (p)ppGpp hydrolase HD (upper part) and synthetase SYNTH (lower part) domains of the RSH1, RSH2, RSH3, and CRSH proteins in *A. thaliana* and *B. napus*. In the HD domain the His (H) and Asp (D), important for its hydrolysis activity, are highlighted in blue. In the SYNTH domain the Gly (G), important for its synthetase activity, is highlighted in pink.

The analysed plant RSH1 proteins contain a functional HD domain, i.e., proteins belonging to this group possess alarmone hydrolytic activity, whereas they do not have a functional (p)ppGpp synthesis domain due to the substitution of functional glycine with serine (Figure 4 and Figure S3). The proteins belonging to RSH2/3 have both (p)ppGpp hydrolase and synthetase activity. CRSH has a functional SYNTH domain, but the hydrolytic domain has lost its activity because of the substitution, conserved in bacterial and plant proteins, of histidine (H) and aspartic acid (D) with serine and glutamic acid, respectively. The *E. coli* RelA protein is also characterized by the lack of a functional HD domain due to the substitution of His and Asp with phenylalanine and proline, respectively (Supplementary Figure S3). The catalytic activity of plant RSH proteins predicted by the in silico analysis of amino acid sequences could be confirmed by a complementation test in *E. coli relA^−^* and *relA^−^*/*spoT^−^* mutants. It was shown that RSH1 proteins from *A. thaliana* and *I. nil* do not possess (p)ppGpp synthase activity, whereas AtRSH2, AtRSH3, and InRSH2, are able to synthesise and hydrolyse alarmones [25,26,41]. The (p)ppGpp synthesis activity was confirmed also for RSH2/3 from *Suaeda japonica* [45], and for *Nicotiana tabacum* RSH2 alarmone synthesis and hydrolysis activity was shown [44]. Interestingly, AtCSRH has only (p)ppGpp synthase activity, as expected based on amino acid sequence analysis [51], whereas InCRSH complements both mutations suggesting that this protein is able also to hydrolyse alarmones, despite the crucial His and Asp in HD domain in InCRSH being substituted with Arg and Gln [25].

Plant RSH proteins such as bacterial Rel, RelA, and SpoT proteins belong to the so-called “long RSH” group. However, there are also “short RSH” proteins containing either a SYNTH domain (SAS) or an HD domain (SAH), without any regulatory domains. SAS and SAH are present in some bacteria together with long RSH. It was hypothesised that “short RSH” proteins allow different lineages of bacteria to expeditiously adapt to fluctuating environments, increasing their chance to survive harsh environmental conditions [36]. In metazoa, SpoT homolog 1 (Mesh) is a class of SAH and contains only (p)ppGpp hydrolytic domains [34]. However, in plants no representatives of “short” RSH proteins have been identified. In some plant species the degradation of the HD domain has been shown, however mostly in algae species [36]. Interestingly, one of the RSH3 proteins in *B. napus* (Figure 3) contains only an HD domain that is an unprecedented feature of plant RSH. However, the functionality of this truncated protein remains to be confirmed. The degradation of the HD or SYNTH domains in plant RSH proteins suggests subfunctionalization similar to that found in bacteria specialised RSHs which may be needed to strengthen the stringent response [24].

Although plant RSHs are nuclear-encoded proteins they contain chloroplast transit peptides at their N-terminus [31]. In silico analysis of putative amino acid sequences of RSH proteins from the *Brassicaceae* family also showed that the chloroplast is the most probable subcellular localisation (Table 1). Interestingly, in the case of CRSH, the presence of a chloroplast signal peptide is less probable than for other types of RSH protein. In fact, the chloroplast localization has been shown for many of these proteins belonging to all types of plant RSH groups [26,31,44,51,52,53]. There is a paucity of reports of the direct measurement of (p)ppGpp in whole plants, and in particular, in isolated chloroplasts. Takahashi et al. [54] showed that the level of ppGpp in pea chloroplasts is 13 times higher than in shoots, which confirmed, that the majority of alarmones in plants are localized in chloroplasts. Later reports have determined the level of (p)ppGpp only in whole plants [41,56,57].

**Figure 5:**
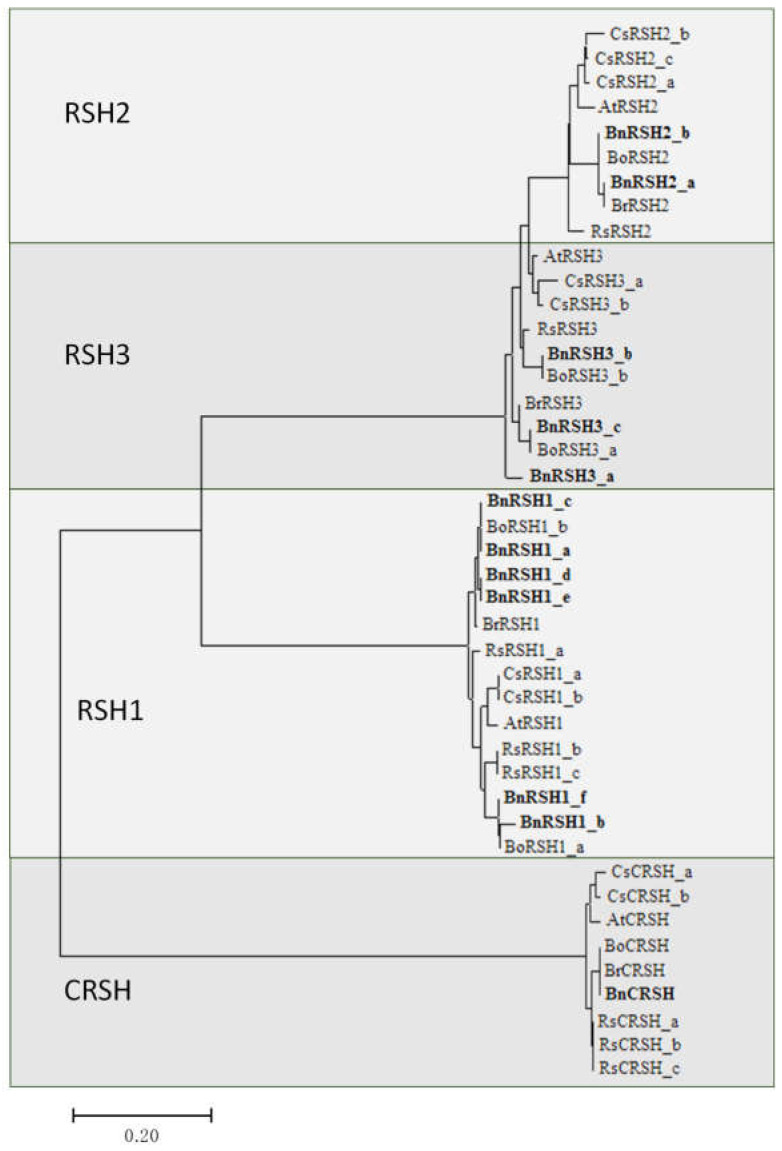
The phylogenetic analysis of RSH proteins based on predicted amino acid sequences given in Table 1. The evolutionary history was inferred using the Neighbor–Joining method by MEGA7.0 software. The optimal tree, with the sum of branch length = 3.01054035, is shown. The tree is drawn to scale, with branch lengths in the same units as those of the evolutionary distances used to infer the phylogenetic tree. The evolutionary distances were computed using the Poisson-correction method and are in the units of the number of amino acid substitutions per site. All positions containing gaps and missing data were eliminated. *B. napus* RSH sequences are indicated in bold.

The phylogenetic analysis of RSH proteins from selected *Brassicaceae* species (Figure 5) showed the presence of three separate RSH groups. RSH2 and RSH3 could be distinguished but, due to sequence similarity, they are grouped on one branch of the phylogenetic tree. In *A. thaliana*, true *RSH3* homologs are missing since AtRSH2 and AtRSH3 are the result of recent duplication of the ancestral *RSH2* gene with a 75% amino acid sequence similarity [36]. True RSH3 homologs are, however, present in other plants. Interestingly, the amino acid sequence similarities between RSH2 and RSH3 in other plant species analysed in this study are very high (ranging from 74% to even 80%), which may suggest that, similar to *A. thaliana*, a true RSH3 homolog is also missing from other plants belonging to the *Brassicaceae* family.

### 2.2. Regulatory Elements Present in Promoter Regions of B. napus RSH Genes

The expression of plant *RSH* genes is tissue/organ-dependent; it depends on the stage of development as well as on the type of the *RSH* gene. It is generally thought, that (p)ppGpp affects gene expression in chloroplasts at transcriptional, translational, and post-translational level, and thus alarmones regulate plant growth and development, and response to stress stimuli [55]. In fact, the expression of *RSH* genes, and thus the level of alarmones, is up-regulated by different factors, including abscisic acid [53,56], salt stress [5,25,26,59], oxidative stress [57], drought [25], and the presence of plant growth promoting bacteria [5]. Interestingly, it was also shown that the overaccumulation of (p)ppGpp in plants has some negative effects. For instance, *Arabidopsis* plants overexpressing *RSH2* and *RSH3* were smaller, contained less chlorophyll, and their seeds had lower vigour [41]. The increased level of (p)ppGpp in *Arabidopsis* led to dwarf chloroplasts, and reduction of metabolites, however, the mutant plants were more tolerant to nutrient-deficient conditions than wild-type plants [52]. Moreover, the increased level of alarmones increased the susceptibility of plants to turnip mosaic virus, whereas for plants with a decreased level of (p)ppGpp, reduced susceptibility was observed [58]. These results clearly show that the level of (p)ppGpp is tightly controlled, since alarmones are critical not only for plastid development and metabolism, but also for the fine-tuning of plant growth and development.

**Figure 6:**
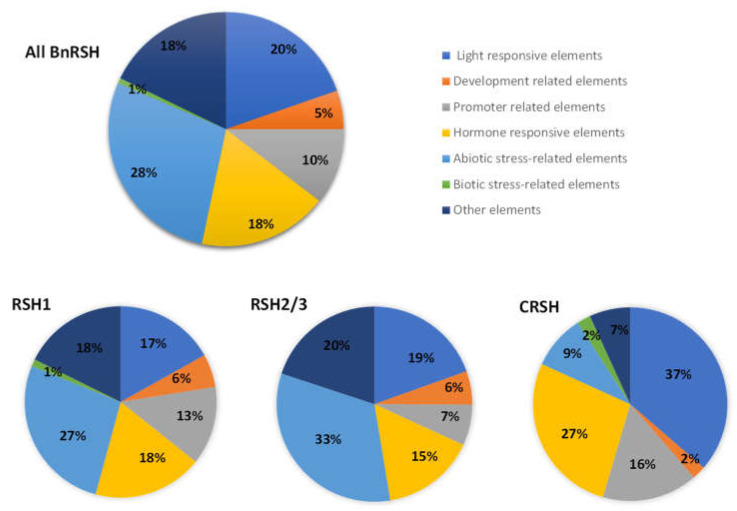
Frequencies of putative *cis*-regulatory elements in *B. napus RSH* genes (upper pie chart), and in *BnRSH1*, *BnRSH2/3*, and *CRSH* genes (lower pie charts). Pie charts depict the *cis*-regulatory elements categorized in seven types according to their predicted functions.

Promoters are responsible for controlling the efficiency, timing, and location of gene expression via clusters of short sequences, including *cis*-regulatory elements (CREs). CREs provide binding sites for transcription factors [37,62,63] and their presence may reflect multiple pathways of gene expression regulation. In order to gain some insight into the putative roles of *BnRSH*, in silico analysis of promoter regions, using the PlantCare database, was performed. This kind of bioinformatical analysis provides a background for further research [59,60,61,62]. A promoter analysis of the *BnRSH* genes revealed the presence of several putative *cis*-acting elements involved in light signalling, in plant development, in response to plant hormones, as well as in plant response to abiotic and biotic stress (Supplementary Table S1). The most abundant elements in all *BnRSH* genes were those related to the abiotic stress response, followed by light- and hormone-responsive elements. Only 1% of all identified CREs in *BnRSH* genes were related to the biotic stress response, and this kind of element was not identified in *BnRSH2/3* genes (Figure 6 and Supplementary Table S2). The highest number of elements was identified in the *BnRSH3_b* gene (69), and the lowest in the *BnRSH1_b* gene (25) (Supplementary Table S1 and Supplementary Figure S4). The most abundant of the abiotic stress response elements were the drought and ABA response element MYB (41), followed by MYC (27), which is a drought, ABA, and cold response element, and the general stress-response element, STRE (22). Among hormone-responsive elements the ethylene response element was the most frequently occurring (36) (Supplementary Table S1). The frequencies of the types of CRE in *BnCRSH* genes were different from the frequencies observed in *BnRSH1–3* genes. In the promoter region of *BnCRSH*, the most abundant elements were those related to response to light, followed by hormone responsive elements. Only 9% of CREs were abiotic stress response elements (Figure 6). This observation may imply that CRSH plays a significantly different physiological role than RSH1–3. In fact, the expression of *CRSH* was not changed by salt stress, osmotic stress, or drought in *I. nil* [25]. The expression of *AtCRSH* was also stable in response to wounding and NaCl, however, it was also not changed by hormones, even ABA [26], and the ABA-response element is the most abundant among hormone responsive elements in the *BnCRSH* gene promoter (Supplementary Table S1). Interestingly, the circadian rhythm of *AtCRSH* expression is also different to that of *AtRSH1–3*, i.e., the expression peak of *AtCRSH* is during darkness whereas *AtRSH1–3* genes are mostly expressed in the light [26].

The presence of multiple putative regulatory elements involved in the light response in promoter regions of *BnRSH* genes suggests that the potential roles of corresponding proteins may not be restricted to the stress response but are also important for plant growth and developmental programs. Additionally, promoters of *BnRSH1_a*, *BnRSH1_c*, *BnRSH1_d*, *BnRSH1_e*, and *BnCRSH* genes contain motifs involved in the control of the circadian cycle (Supplementary Table S1). It was shown that the mRNA level of *RSH* genes and alarmone levels are light dependent. The expression of all *RSH* genes in *Arabidopsis* fluctuated during the diurnal time course [26]. Takahashi et al. [54] showed that prolonged darkness (12 h) reduced ppGpp levels, whereas abrupt changes to *Pisum sativum* plants, from prolonged light (12 h) to dark, caused a substantial elevation in ppGpp levels. Similarly, alarmone concentration altered in 12-h light/12-h dark cycling conditions, with increasing alarmone levels at the beginning, and its highest peak during the dark time period [63]. The functionality of the identified potential *cis*-elements needs to be further confirmed.

### 2.3. Effect of Salinity and Rhizobacteria on the Expression of BnRSH Genes

Soil salinity stress mitigates crop productivity and is an important challenge for global sustainable agriculture [64]. It affects several aspects of plant metabolism leading to significant decreases in plant growth and yield [6]. *B. napus* is considered one of the most saline-resistant species in the genus *Brassica*, being more tolerant not only than its diploid ancestors, but also than other polyploid species [65]. Salinity had a visible impact on *B. napus* seed germination (Supplementary Figure S5) and the growth of 6-day-old rapeseed seedlings (Supplementary Figure S6). The germination ratio was visibly decreased even in 50 mM NaCl whereas in the presence of 200 mM NaCl less than half of the seeds germinated in comparison to the control (seeds germinated in water). The length of root and hypocotyl, as well as the fresh and dry biomass of *B. napus* seedlings, significantly decreased in the presence of salt (Supplementary Figure S6) and the most affected by NaCl was hypocotyl growth (Supplementary Table S3).

**Figure 7:**
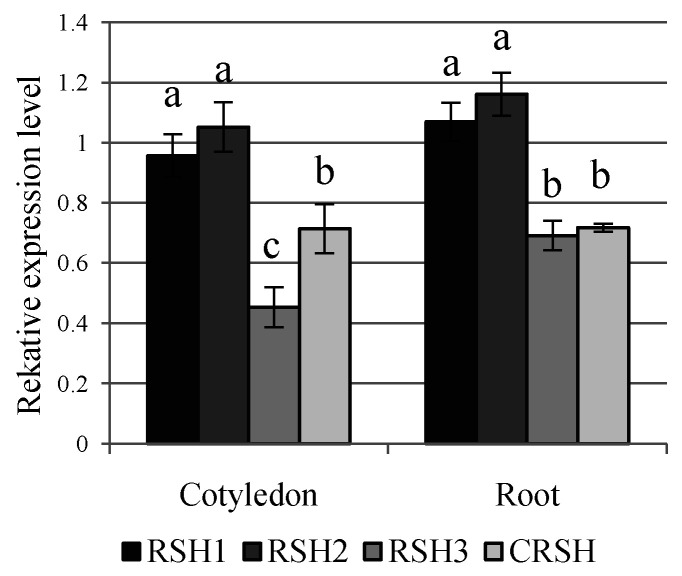
Expression analysis of *BnRSH* in the *B. napus* cotyledons and roots of 6-day-old seedlings. Chart shows the relative transcript level of analysed genes (*BnRSH1_b*, *BnRSH2_b*, *BnRSH3_a*, and *BnCRSH*) with respect to the expression of the reference gene (*BnAc*). Different letters indicate statistically significant changes according to one-way ANOVA test at *p* < 0.05. Bars represents means ± SD.

**Figure 8:**
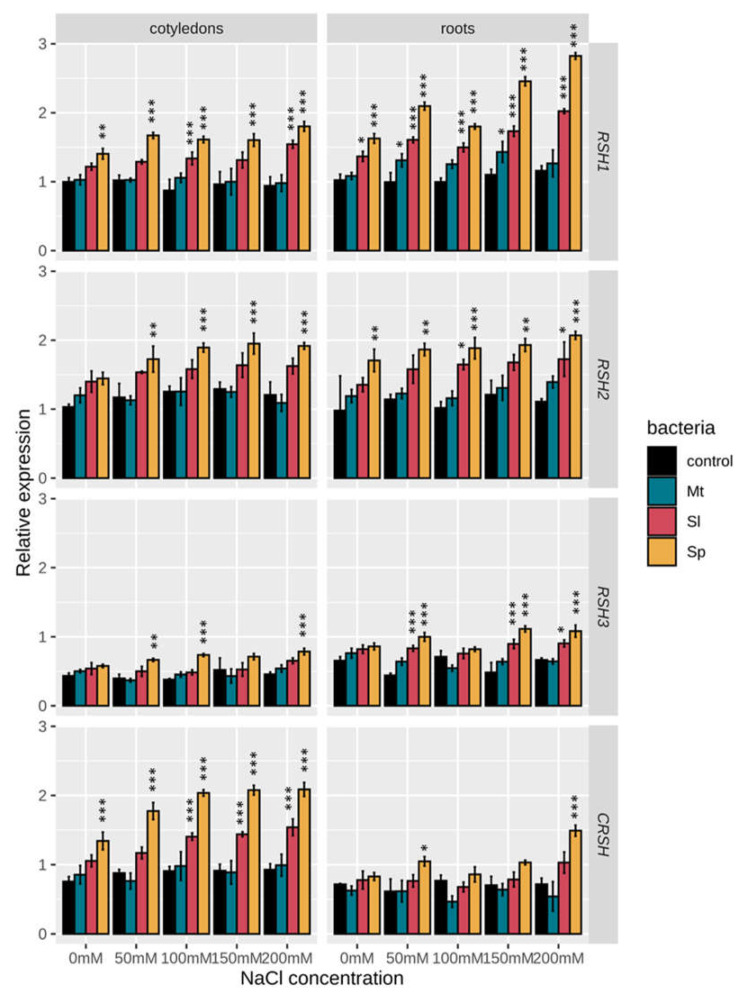
Expression analysis of *BnRSHs* in salt stress and in the presence of PGPR bacteria using sqRT-PCR. Charts show the relative transcript level of *BnRSH1_b*, *BnRSH2_b*, *BnRSH3_a*, and *BnCRSH* genes with respect to the expression of a reference gene (*BnAc*). Bars represent means ± SD. Control (black bars) are plants grown in in different NaCl concentrations (0 mM, 50 mM, 100 mM, 150 mM, and 200 mM NaCl) but without inoculation with bacteria. Mt (green bars)—*M. timonae*, Sl (red bars)—*S. liquefaciens*, Sp (yellow bars)—*S. plymuthica*. Statistical analysis was performed using two-way ANOVA followed by Scheffe post-hoc test. Asterisks indicate statistically significant differences in comparison to the control (i.e., expression of a particular *RSH* gene in plants grown in the same concentration of salt but without bacteria—black bars) at *p*-value < 0.001 (***), *p*-value < 0.01 (**), and *p*-value < 0.05 (*). Full statistical analysis data are available as Supplementary Materials (Supplementary Tables S4–S11).

The potential involvement of *RSH* genes and alarmones in the plant response to salt stress has been shown previously [26,43,44,60]. In order to gain more insight into the possible physiological roles of *BnRSHs*, the expression of four selected *B. napus RSH* genes (*RSH1_b*, *RSH2_b*, *RSH3_a*, *CRSH*) was analysed using sqRT-PCR in seedling organs (Figure 7) in response to salt stress, and in response to the presence of PGPR bacteria (Figure 8). *BnRSH* genes were differentially expressed in cotyledons and roots, i.e., *BnRSH1* and *BnRSH2* genes were highly expressed, while *BnRSH3* and *BnCRSH* mRNAs were expressed at a lower level in both organs.

Using histochemical staining of GUS activity, it was shown that in *Arabidopsis*, *AtRSH1* and *AtRSH3* were highly expressed in hypocotyls and leaves, whereas *AtRSH2* and *AtCRSH* were expressed in leaves. In the roots of seedlings only *AtRSH2* was expressed, whereas in the roots of mature plants, *AtRSH3* was also expressed [26]. Using RT-PCR, high expression of *AtRSH1* and *AtRSH3*, and low expression of *AtRSH2* in shoots, were also shown. In the roots, *AtRSH2* and *AtRSH3* were highly expressed, whereas *AtRSH1* was expressed at a low level. *AtCRSH* was not tested in this study [66]. In rice, *OsCRSH* was expressed both in roots and shoots, however, in roots at a lower level than in shoots [31]. In contrast, in the cotyledons of *I. nil* seedlings, *RSH1*, *RSH2*, and *CRSH* were equally highly expressed, whereas in roots, *RSH2* was highly expressed, *RSH1* was expressed at the low level, and no expression of *CRSH* was detected [25].

In general, salinity stress had no significant effect on the expression of *BnRSH* genes in cotyledons and roots (Figure 8). The levels of *BnRSH2* and *BnCRSH* transcripts in cotyledons, and the levels of *BnRSH1* and *BnCRSH* in roots, slightly increased under salinity stress as compared with control plants, and the differences were statistically significant (Supplementary Tables S5, S6, S10 and S11). Interestingly, previous studies showed that *A. thaliana* treated with 250 mM NaCl exhibited increased *AtRSH2* expression, but that salt had no impact on the expression of *AtRSH1*, *AtRSH3*, and *AtCRSH* [26], whereas, in another study, treatment with 250 mM NaCl significantly increased both *AtRSH2* and *AtRSH3* transcript levels, decreased the amount of *AtCRSH* mRNA, and had no impact on *AtRSH1* expression [57]. Similarly, Prusińska et al. [25] showed that salt stress (300 mM NaCl) elevated the *InRSH2* transcript level, whereas both *InRSH1* and *InCRSH* did not show substantial changes in 5-day-old *I. nil* seedlings. Although, in promoters of *BnRSH* genes, several putative regulatory *cis*-elements involved in response to varied abiotic stresses, possibly including salinity stress, have been identified (Supplementary Table S1), the stable expression of *BnRSHs* in response to salt has been observed. This may be due to the concentrations of NaCl used in this study. Using an NaCl solution, up to 200 mM mimics non saline, slightly saline, and medium saline soils, whereas a concentration above 250 mM is typical for highly saline soils [67]. Moreover, the observed, almost changeless expression of *BnRSH* genes in response to NaCl, and the differences in expression of *RSH* genes response to salinity among plants, might be caused by the different developmental stages of the analysed plants, and/or varied sampling time points.

The effects of the rhizobacteria, *S. plymuthica*, *S. liquefaciens*, and *M. timonae*, on the expression of *BnRSHs* in leaves and roots was investigated (Figure 8). Using plant growth- promoting bacteria to improve plant tolerance to environmental stresses, including salt stress, in order to obtained a high yield even in adverse environmental conditions, is considered an economically and environmentally friendly approach [9]. Earlier reports have shown that PGPR bacteria mitigate salt stress via varied mechanisms including the production of indole acetic acid (IAA) [6], induction of potassium and calcium accumulation in plants, increased content of osmolytes including proline [68], and activation of plant antioxidant enzymes [69]. Using two-way ANOVA, significant interactions between salt concentration and species of bacteria for all analysed genes, besides *BnRSH2* in roots, has been found (Supplementary Tables S4–S11). Therefore, we examined the bacteria simple main effect, i.e., the differences between the expression of *BnRSHs* in plants inoculated with different bacteria, for each salt concentration. Among all analysed bacteria *S. plymuthica* had the greatest impact on the expression of all *BnRSH* genes in all tested salt concentrations, both in cotyledons and roots. The expression of *BnRSH1* was upregulated by *S. plymuthica* and *S. liquefaciens* in both cotyledons and roots, whereas *M. timonae* increased the expression of *BnRSH1* in roots only (Figure 8). *S. plymuthica* increased the expression of *BnRSH2* and *BnRSH3* in cotyledons and roots, while *S. liquefaciens* increased the expression of *BnRSH2* in roots only. The expression of *BnCRSH* in roots is mostly unaffected by PGPR bacteria, whereas *S. plymuthica* and *S. liquefaciens* induced the expression of *BnCRSH* in cotyledons (Figure 8). In response to salt stress, *BnRSH* gene expression is elevated in *S. plymuthica* and *S. liquefaciens* inoculated plants, whereas *M. timonae* inoculated plants did not show substantial changes as compared with control plants (without bacteria but treated with NaCl at the same concentration). For all *BnRSH* genes the highest level of expression was observed in plants inoculated with *S. plymuthica* (Figure 8). There is little data in the literature about the possible relation between (p)ppGpp and PGPR bacteria. Szymańska et al. [5] showed changes in the expression of *BnRSH1* and *BnRSH3* in roots of oilseed rape growing in the presence of the halotolerant PGPR bacterium *Pseudomonas stutzeri* ISE12 under salt stress. Increased expression of plant *RSH* genes was also demonstrated in response to pathogen attack. It was found that the infection of tobacco plants with the bacterial *Erwinia carotovora* pathogen leads to a 10-fold increase in the *NtRSH2* protein level [44].

*S. plymuthica* used in this study is characterized by high metabolic activity; it is able to biodegrade plastic in compost and agricultural soil and stimulate the growth of *B. napus*, *Miscanthus x giganteus*, and *Salix viminalis* [70,71]. It was shown that several salt-tolerant strains of *S. plymuthica* improved cucumber biomass and yield via synthesis of IAA [72]. *S. liquefaciens* improved salt stress tolerance and plant growth in maize and rape [6]. *M. timonae* colonizes the rhizosphere, roots and leaves, and is a growth promoter via the production of IAA and siderophores in various plant species [73]. Our research clearly showed changes in mRNA levels of *BnRSHs* grown in the presence of the strains *S. liquefaciens* and *S. plymuthica*, but not in the presence of *M. timonae* which suggests that some PGPR bacteria might also improve plant growth under salt stress via the stringent response pathway.

## 3. Materials and Methods

### 3.1. In Silico Analysis of B. napus, B. olearacea, B. rapa, C. sativa, and R. sativus RSH Genes and Proteins

The *RSH1*, *RSH2*, *RSH3,* and *CRSH* in the plant genomes selected for this study from *Brassicaceae* family genes have been identified using *A. thaliana RSH* cDNA sequences (*AtRSH1*, *AtRSH2*, *AtRSH3*, and *AtCRSH*) as queries. A search was performed using BLASTN (Basic Local Alignment Search Tool) using the NCBI (ncbi.nlm.nih.gov, accessed on 10 May 2021) nucleotide database. The analysis of the intron-exon organisation was carried out using the CIWOG tool (http://peroxibase.toulouse.inra.fr/tools/ciwog_search_form, accessed on 15 May 2021) [74]. The putative amino acid sequences were then obtained from the NCBI protein database. For primary and secondary structure predictions of RSH proteins InterProScan (https://www.ebi.ac.uk/interpro/search/sequence/, accessed on 23 May 2021), Conserved Domain Search (https://www.ncbi.nlm.nih.gov/Structure/cdd/wrpsb.cgi, accessed on 23 May 2021), and PSIPRED (http://bioinf.cs.ucl.ac.uk/psipred/, accessed on 24 May 2021) were utilized. Clustal Omega was used for multiple sequence alignments (http://www.clustal.org/omega/, accessed on 27 May 2021) [75]. For calculation of molecular mass and pI of putative RSH proteins the Compute pI/Mw tool (https://web.expasy.org/compute_pi/, accessed on 13 June 2021) was utilised. TargetP (http://www.cbs.dtu.dk/services/TargetP/, accessed on 14 June 2021) [76,77] was used to predict subcellular localization of analysed RSH proteins. The phylogenetic analysis was caried out in MEGA7 software [78,79] using the neighbour-joining method [80].

The promoter regions of *BnRSH* genes were analysed using the PlantCARE database (http://bioinformatics.psb.ugent.be/webtools/plantcare/html/, accessed on 10 May 2021) [59]. For each *BnRSH* gene a 1500-bp long fragment including promoter and 5′UTR of genomic DNA was retrieved from the NCBI GenBank (http://www.ncbi.nlm.nih.gov/genbank/, accessed on 5 May 2021).

### 3.2. Bacterial Strains

Three bacterial strains: *Massilia timonae* [81], *Serratia liquefaciens* [82], and *Serratia plymuthica* [71,83], obtained from the collection of Professor Katarzyna Hrynkiewicz from the Department of Microbiology at the Nicolaus Copernicus University in Toruń, were used in the experiments. Bacteria were grown in R2A (Difco, Franklin Lakes, NJ, USA) liquid medium (18 g/L) at 24 °C for 24 h. The optical density of bacterial culture was checked spectrophotometrically at λ = 600 nm (SmartSpec Plus, BioRad, Hercules, CA, USA) and adjusted to the value of 5 × 10^6^ c.f.u./cm^3^ [8].

### 3.3. Plant Material

Seeds of the *B. napus* L. winter cultivar ‘Harry’ (Obrol Company, Kruszewnia, Poland) were surface sterilized with a mixture of 30% hydrogen peroxide and 96% ethanol (1:1, *v*/*v*) for 3 min and rinsed at least six times with sterile distilled water. The seeds were inoculated with a bacterial suspension, prepared as described above, and incubated for 10 min, with shaking, at room temperature. Non-inoculated (control) and inoculated seeds were placed in Petri dishes on filter paper moistened with 5 mL of sterile water (control) and 50, 100, 150, and 200 mM NaCl.

To analyse the impact of NaCl on *B. napus* seed germination and seedling growth, seeds were incubated in 16 h darkness/8 h light photoperiod at 24 °C for 6 days. The number of germinated seeds was checked after 14 h, 17 h, 20 h, 24 h, and 48 h of the start of experiment. The length of the hypocotyl and roots of 6-day-old seedlings were measured. Moreover, the fresh mass of 10 6-day-old seedlings was determined, and after drying (80 °C for 24 h) the dry mass of 10 seedlings was determined.

For *BnRSH* gene expression analysis, seeds were incubated for a 16 h darkness/8 h light photoperiod at 24 °C for 6 days. Cotyledons and roots of 6-day-old seedlings were frozen in liquid nitrogen and stored at −80 °C until RNA isolation was performed. The experiments were performed in triplicates.

### 3.4. Expression Analysis of BnRSH Genes

Total RNA was extracted from the *B. napus* organs using TRI Reagent (Sigma-Aldrich, Poznań, Poland), according to the manufacturer’s protocol. RNA was analysed by spectrophotometric measurement and gel electrophoresis in 1% agarose gel in 1x TAE (Tris-Acetate-EDTA) buffer stained with ethidium bromide. Prior cDNA synthesis from 1 μg of RNA genomic DNA was removed using RNAse free DNase I (Thermo Fisher Scientific, Waltham, MA, USA). Further oligo(dT)_18_ primer and RevertAid reverse transcriptase (Thermo Fisher Scientific, Waltham, MA, USA) were used for cDNA synthesis, in accordance with the protocol described in [84].

Semi-quantitative RT-PCR (sqRT-PCR) assays were performed to evaluate the effects of NaCl and/or the presence of PGPR on mRNA level of *RSH* genes. For each pair of primers, the PCR conditions, including the concentration of primers, DNA polymerase, and Mg^2+^, annealing temperature, and the number of cycles, were optimised according to [85]. The relative expression level of *BnRSH1*, *BnRSH2*, *BnRSH3*, and *CRSH* genes, was expressed as a ratio of the amount of PCR product for analysed gene to the amount of PCR product for the reference gene. *B. napus* actin-7 (*BnAc*, NCBI GenBank accession no. XM_013858992.2) was used as a reference gene. The PCR reaction mixture contained: 1.25 U of Opti*Taq* DNA polymerase (EURx, Gdańsk, Poland), 1.5 μL of cDNA as the template, 0.15 µM of each primer, and 1.5 mM MgCl_2_, in a total reaction volume of 20 μL. Primers are listed in Table 2. The thermal cycling conditions were as follows: 95 °C for 30 s, 54 °C (*BnRSH2*, *BnRSH3*), 52 °C (*BnCRSH*), or 58 °C (*BnRSH1*) for 40 s, and 72 °C for 40 s for 26 cycles (*BnAc*), 39xcycles (*BnRSH1*), 33 cycles (*BnRSH2*, *BnRSH3*), and 37 cycles (*BnCRSH*). Products of sqRT-PCR were separated on a 1.5% agarose gel with EtBr in TAE buffer and quantified by intensity using the ImageGauge 3.46. software (FujiFilm, Tokyo, Japan). Each reaction was repeated three times.

### 3.5. Statistical Analysis

Statistical differences of *BnRSH* gene expression data were assessed using one-way ANOVA followed by Tukey’s honest significance test (for comparison of *BnRSHs* expression in cotyledons and roots) or two-way ANOVA test followed by Scheffe post-hoc test (for comparison of *BnRSHs* expression in response to salt, and the presence of PGPR bacteria). Results are means ± SD. For one way ANOVA, a *p*-value < 0.05 was considered statistically significant. For two-way ANOVA, *p*-values < 0.05 (*), < 0.01 (**), and < 0.001 (***), were considered statistically significant. Statistical analyses were performed using R version 4.1.1 and packages DescTools and ggplot2 (r-project.org, accessed on 15 September 2021).

## 4. Conclusions

Our results suggest that in plants belonging to the *Brassicaceae* family the stringent response is coordinated by numerous isoforms of RSH proteins. There is a high level of conservancy between the respective orthologs of *RSH* genes and proteins analysed in the study plant species. Plants possess higher number of genes encoding synthetases and/or hydrolases of alarmones than bacteria, which is especially apparent for polyploid plants, e.g., *B. napus*. The presence of multiple isoforms that underwent subfunctionalization highlights the need of rigorous control of (p)ppGpp-dependent pathways in plants. The mechanisms of the plant stringent response are beginning to emerge, but the specific roles of RSH isoforms are still puzzling. An in silico promoter analysis of *BnRSH* genes revealed the presence of several putative regulatory elements, and indicated that, (i) *RSH* gene expression might be regulated by multiple abiotic and biotic factors, (ii) *RSH* proteins might be involved in varied metabolic pathways, (iii) the possible roles of *RSH1*, *RSH2/3*, and *CRSH*, seems to be diversified. The wet-lab expression analysis of selected *B. napus RSH* genes in response to salt stress supported the idea of different physiological roles of plant RSH isoforms. Moreover, we showed that the plant stringent response might be one of the pathways via which PGPR bacteria promote plant growth and development; however this seems to be bacteria species-dependent.

## Figures and Tables

**Table 1:** *RSH* genes present in the genomes of *A. thaliana*, *B. napus*, *B. oleracea*, *B. rapa*, *C. sativa*, and *R. sativus*. As a comparison, bacterial proteins of the stringent response for *E. coli*, RelA and SpoT, and for *Streptomyces coelicolor*, Rel, were included. The number of exons and introns, the length of CDS, the length, molecular weight, pI, and predicted subcellular localization of putative RSH proteins, are also given. Asterisks (*) indicate the *B. napus* (*RSH1_b*, *RSH2_b*, *RSH3_a*, and *CRSH*) genes that were further analysed for their expression level (vide infra).

Species	Genes	Gene ID	Transcript ID	CDS (bp)	Chromosome Location	Protein ID	AA	pI	Mw (kD)	Introns	Exons	Predicted Transfer Peptide (Probability)
*A. thaliana*	RSH1	828096	NM_116459.4	2655	4	NP_567226.1	883	6.65	98.58	23	24	cTP (0.455), mTP (0.0002), tlTP (0.0051), other (0.5393)
	RSH2	820619	NM_112259.5	2130	3	NP_188021.1	709	6.89	79.05	5	6	cTP (0.6081), mTP (0.0003), tlTP (0.0846), other (0.3047)
	RSH3	841853	NM_104291.8	2148	1	NP_564652.2	715	6.66	79.72	5	6	cTP (0.7887), mTP (0.0024), tlTP (0.0397), other (0.1669)
	CRSH	821012	NM_001338291.1	1752	3	NP_001327079.1	598	6.14	68.28	3	4	cTP (0.0708), mTP (0.1949), tlTP (0.0002), other (0.7341)
*B. napus*	RSH1_a	106345251	XM_013784481.2	2652	unknown	XP_013639935.1	883	6.64	98.56	23	24	cTP (0.3786), mTP (0.0002), tlTP (0.0059), other (0.6149)
	RSH1_b *	106399012	XM_013839498.2	2565	A5	XP_013694952.1	854	6.60	95.88	22	23	cTP (0.4978), mTP (0.0025), tlTP(0.004), other (0.4957)
	RSH1_c	106436227	XM_013877186.2	2652	A8	XP_013732640.1	883	6.64	98.58	23	24	cTP (0.3786), mTP (0.0002), tlTP(0.0059), other (0.6149)
	RSH1_d	106365508	XM_013804925.2	2652	A9	XP_013660379.1	883	6.48	98.55	23	24	cTP (0.5232), mTP (0.0005), tlTP(0.0171), other (0.459)
	RSH1_e	106362473	XM_013802370.2	1860	A9	XP_013657824.1	619	6.62	69.26	19	20	cTP (0.5232), mTP (0.0005), tlTP(0.0171), other (0.459)
	RSH1_f	106381614	XM_013821535.2	2640	C2	XP_013676989.1	879	6.60	98.44	23	24	cTP (0.2021), mTP (0.0011), tlTP(0.0043), other (0.7924)
	RSH2_a	106452255	XM_013894318.2	2055	A5	XP_013749772.2	684	6.67	77.13	5	6	cTP (0.2425), mTP (0.0001), tlTP(0.0058), other (0.7498)
	RSH2_b *	111206471	XM_022703426.1	2091	C5	XP_022559147.1	696	6.56	77.98	5	6	cTP (0.4948), mTP (0.0003), tlTP(0.0816), other (0.4229)
	RSH3_a *	106345829	XM_013785013.2	861	A6	XP_013640467.1	286	6.04	31.11	1	2	cTP (0.6255), mTP (0.0001), tlTP(0.0181), other (0.3557)
	RSH3_b	106431664	XM_013872470.2	2133	C6	XP_013727924.1	710	6.50	78.78	5	6	cTP (0.7621), mTP (0.0005), tlTP(0.0889), other (0.1473)
	RSH3_c	106348454	XM_022704818.1	2109	C6	XP_022560539.1	702	6.77	78.07	6	7	cTP (0.5288), mTP (0.0001), tlTP(0.0244), other (0.446)
	RSH3 _pseudo	106345828	-	-	A6	-	-	-	-	-	-	-
	CRSH *	106389210	XM_013829418.2	1743	C3	XP_013684872.1	580	6.03	65.83	3	4	cTP (0.2211), mTP (0.0648), tlTP(0.0095), other (0.7045)
	CRSH _pseudo	106439579	-	-	A3	-	-	-	-	-	-	-
*B. oleracea*	RSH1_a	106327624	XM_013765826.1	2628	C2	XP_013621280.1	875	6.52	97.87	23	24	cTP (0.1379), mTP (0.0016), tlTP(0.0142), other (0.8462)
	RSH1_b	106318815	XM_013756949.1	2652	C9	XP_013612403.1	883	6.64	98.55	23	24	cTP (0.3786), mTP (0.0002), tlTP(0.0059), other (0.6149)
	RSH2	106295267	XM_013731123.1	2091	C5	XP_013586577	696	6.56	77.98	5	6	cTP (0.4948), mTP (0.0003), tlTP(0.0816), other (0.4229)
	RSH3_a	106300657	XM_013736852.1	2109	C6	XP_013592306.1	702	6.77	78.07	5	6	cTP (0.5288), mTP (0.0001), tlTP(0.0244), other (0.446)
	RSH3_b	106300381	XM_013736509.1	2133	C6	XP_013591963.1	710	6.50	78.78	5	6	cTP (0.7621), mTP (0.0005), tlTP(0.0889), other (0.1473)
	CRSH	106334911	XM_013773298.1	1743	C3	XP_013628752.1	580	6.11	65.88	2	3	cTP (0.2263), mTP (0.0536), tlTP(0.0104), other (0.7096)
*B. rapa*	RSH1	103836764	XM_033278751.1	2685	A9	XP_033134642.1	894	6.38	100.02	23	24	cTP (0.5255), mTP (0.0005), tlTP(0.0172), other (0.4566)
	RSH2	103870072	XM_009148172.3	2064	A5	XP_009146420.1	687	6.67	77.3	5	6	cTP (0.2318), mTP (0.0001), tlTP(0.0048), other (0.7612)
	RSH3	103871068	XM_009149293.3	2091	A6	XP_009147541.1	696	6.30	77.87	5	6	cTP (0.6833), mTP (0.0001), tlTP(0.0135), other (0.3026)
	CRSH	103859710	XM_009137283.3	1731	A3	XP_009135531.1	576	5.99	65.47	2	3	cTP (0.3013), mTP (0.0173), tlTP(0.0221), other (0.6593)
*C. sativa*	RSH1_a	104747094	XM_010468670.2	2664	2	XP_010466972.1	887	6.66	98.83	24	25	cTP (0.5423), mTP (0.0003), tlTP(0.0096), other (0.4475)
	RSH1_b	104737555	XM_010457755.2	2655	13	XP_010456057.1	884	6.56	98.56	25	26	cTP (0.3518), mTP (0.0012), tlTP(0.0168), other (0.6298)
	RSH1 _pseudo	104707874	-	-	8	-	-	-	-	-	-	-
	RSH2_a	104778842	XM_010503267.2	2154	1	XP_010501569.1	717	6.57	79.77	6	7	cTP (0.4377), mTP (0.0002), tlTP(0.0256), other (0.5339)
	RSH2_b	104788263	XM_010513997.2	630	5	XP_010512299.1	209	7.72	23.53	3	4	cTP (0), mTP (0), tlTP(0), other (0.9999)
	RSH2_c	104745674	XM_010466982.2	2148	15	XP_010465284.1	715	6.42	79.56	6	7	cTP (0.3945), mTP (0.0001), tlTP(0.0761), other (0.5286)
	RSH3_a	104778355	XM_010502782.2	2151	3	XP_010501084.1	716	6.19	80.09	5	6	cTP (0.8885), mTP (0.002), tlTP(0.0436), other (0.0634)
	RSH3_b	104758764	XM_010481702.2	2151	17	XP_010480004.1	716	6.77	79.75	5	6	cTP (0.7837), mTP (0.0027), tlTP(0.0167), other (0.1939)
	RSH3 _pseudo 1	104742935	-	-	14	-	-	-	-	-	-	-
	RSH3 _pseudo 2	104761544	-	-	18	-	-	-	-	-	-	-
	CRSH_a	104782095	XM_010506922.2	1764	1	XP_010505224.1	587	6.20	66.97	3	4	cTP (0.2705), mTP (0.0903), tlTP(0.0037), other (0.6353)
	CRSH_b	104765592	XM_010489335.2	1758	19	XP_010487637.1	585	6.07	66.89	3	4	cTP (0.0869), mTP (0.1501), tlTP(0.0008), other (0.7621)
*R. sativus*	RSH1_a	108828360	XM_018602017.1	2640	unknown	XP_018457519.1	879	6.78	97.76	23	24	cTP (0.6503), mTP (0.0013), tlTP(0.0287), other (0.3196)
	RSH1_b	108843457	XM_018616659.1	2601	unknown	XP_018472161.1	866	6.96	97	23	24	cTP (0.1051), mTP (0.0009), tlTP(0.0005), other (0.8934)
	RSH1_c	108834481	XM_018607822.1	1290	unknown	XP_018463324.1	429	7.56	48.26	13	14	cTP (0.1051), mTP (0.0009), tlTP(0.0005), other (0.8934)
	RSH2	108863143	XM_018637469.1	2037	unknown	XP_018492971.1	678	6.55	76.31	5	6	cTP (0.2086), mTP (0), tlTP(0.0096), other (0.7815)
	RSH3	108862601	XM_018636787.1	2121	unknown	XP_018492289.1	706	6.44	78.36	6	7	cTP (0.6764), mTP (0.0001), tlTP(0.1784), other (0.1413)
	RSH3 _pseudo	108815328	-	-	unknown	-	-	-	-	-	-	-
	CRSH_a	108857634	XM_018631638.1	1749	unknown	XP_018487140.1	582	6.06	66.11	3	4	cTP (0.1098), mTP (0.0301), tlTP(0.0031), other (0.857)
	CRSH_b	108857621	XM_018631622.1	1749	unknown	XP_018487124.1	582	6.06	66.11	3	4	cTP (0.1098), mTP (0.0301), tlTP(0.0031), other (0.857)
	CRSH_c	108857284	XM_018631245.1	1749	unknown	XP_018486747.1	582	6.06	66.08	3	4	cTP (0.1098), mTP (0.0301), tlTP(0.0031), other (0.857)
*E. coli*	RelA	947244	-	2235	-	NP_417264.1	744	6.29	83.89	-	-	-
	SpoT	948159	-	2109	-	NP_418107.1	702	8.89	79.34	-	-	-
*S. coelicolor*	Rel	1096939	-	2544	-	WP_003977314.1	847	9.36	94.2	-	-	-

Gene ID, transcript ID, protein ID—accession numbers from NCBI GenBank, cTP—chloroplast transit peptide, mTP—mitochondrial transit peptide, tlTP—tonoplast transit peptide, other—most probable cytoplasmic protein.

**Table 2:** Sequences of primers used for expression analysis of *B. napus RSH* genes.

Primer Name	Sequence of Primers 5′–3′	Analysed Gene and Amplicon Length [bp]
BnRSH1_f BnRSH1_r	GGAGGTTCAGATCAGAACGG CCATTCACCTTCGCTGCTAC	*BnRSH1* 396
BnRSH2_f BnRSH2_r	GCAAGATGTTGAAGAATCTAACG GCACAGACATCTTGTCATTTTCG	*BnRSH2* 534
BnRSH3_f BnRSH3_r	CCGAAACTTTCCGATTTCAA TCGTAGTCAACGCACGAGTC	*BnRSH3* 524
BnCRSH_f BnCRSH_r	AAGTGATGGAGGAGCTTGGA CCATTTACTGGAACGCAACA	*BnCRSH* 263
BnAc_f BnAc_r	CTCACGCTATCCTCCGTCTC TTGATCTTCATGCTGCTTGG	*BnAc* 469

## Data Availability

Not applicable.

## Supplementary Materials

The following are available online at https://www.mdpi.com/article/10.3390/ijms221910666/s1.
