# Supplementary material for: In Silico Study of the RSH (RelA/SpoT Homologs) Gene Family and Expression Analysis in Response to PGPR Bacteria and Salinity in Brassica napus
Source: Int J Mol Sci. 2021 Oct 1;22(19):10666. doi: 10.3390/ijms221910666 (PMC8509286; doi:10.3390/ijms221910666)
Supplement: Supplementary file 1 [file ijms-22-10666-s001.zip › Dabrowska et al. suppl figs.pdf]

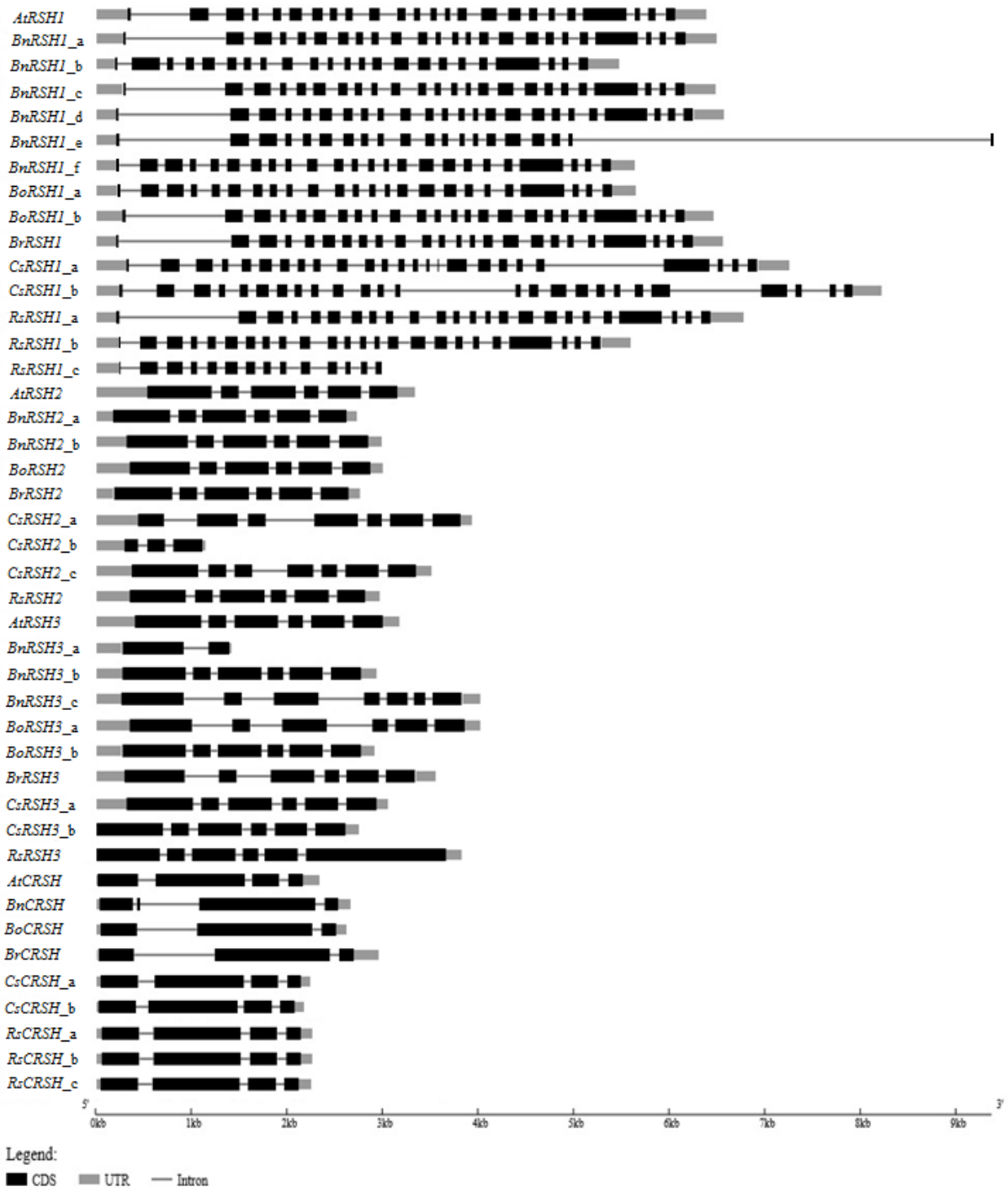

Supplementary Figure S1. Intron-exon structure of *RSH* genes in *A. thaliana*, *B. napus*, *B. olearacea*, *B. rapa*, *C. sativa* and *R. sativus*. Grey rectangles indicate UTRs, and black rectangles indicate coding sequence. Intron positions are marked by lines.

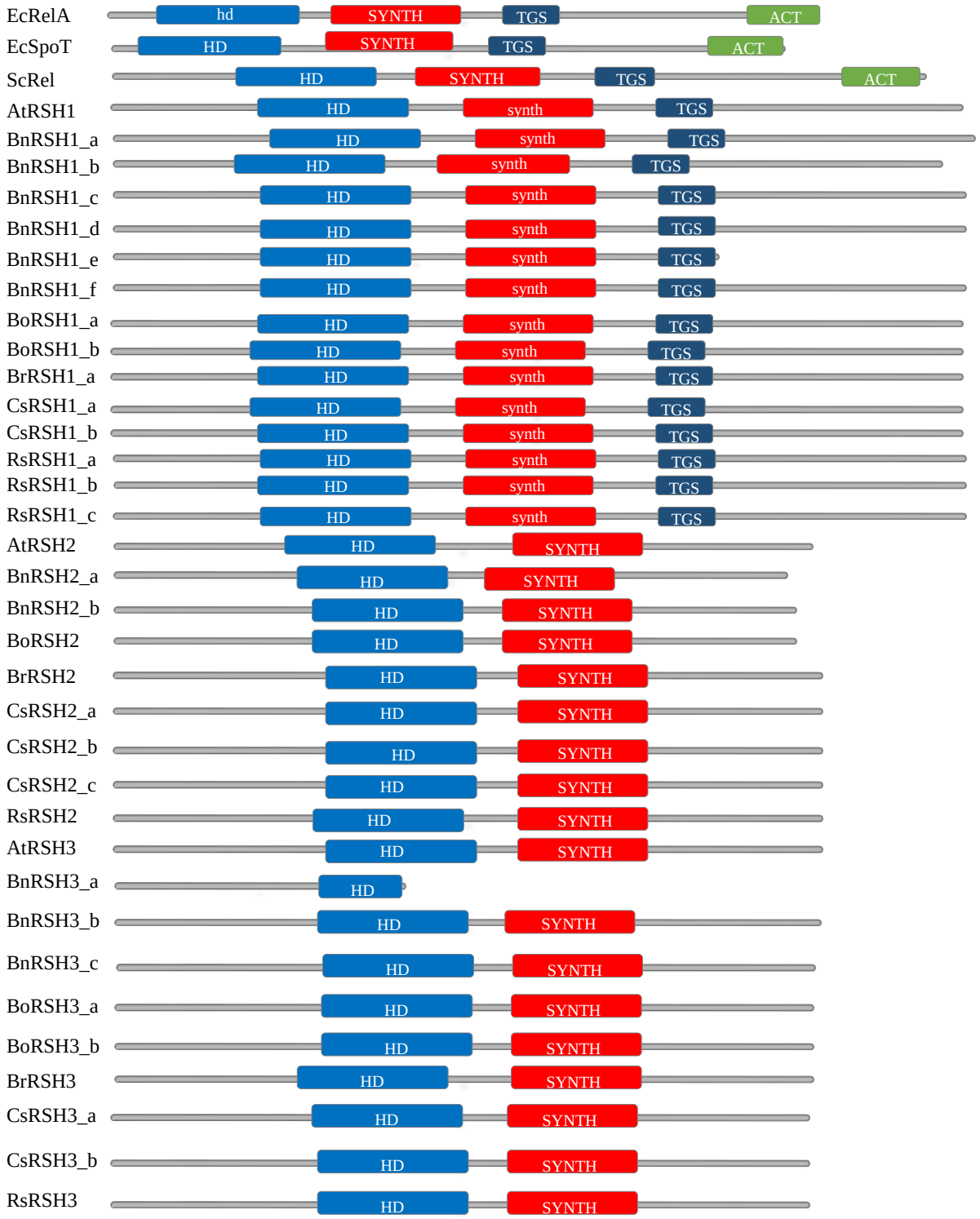
EcRelA
hd
SYNTH
TGS
ACT
EcSpoT
HD
SYNTH
TGS
ACT
ScRel
HD
SYNTH
TGS
ACT
AtRSH1
HD
synth
TGS
BnRSH1_a
HD
synth
TGS
BnRSH1_b
HD
synth
TGS
BnRSH1_c
HD
synth
TGS
BnRSH1_d
HD
synth
TGS
BnRSH1_e
HD
synth
TGS
BnRSH1_f
HD
synth
TGS
BoRSH1_a
HD
synth
TGS
BoRSH1_b
HD
synth
TGS
BrRSH1_a
HD
synth
TGS
CsRSH1_a
HD
synth
TGS
CsRSH1_b
HD
synth
TGS
RsRSH1_a
HD
synth
TGS
RsRSH1_b
HD
synth
TGS
RsRSH1_c
HD
synth
TGS
AtRSH2
HD
SYNTH
BnRSH2_a
HD
SYNTH
BnRSH2_b
HD
SYNTH
BoRSH2
HD
SYNTH
BrRSH2
HD
SYNTH
CsRSH2_a
HD
SYNTH
CsRSH2_b
HD
SYNTH
CsRSH2_c
HD
SYNTH
RsRSH2
HD
SYNTH
AtRSH3
HD
SYNTH
BnRSH3_a
HD
BnRSH3_b
HD
SYNTH
BnRSH3_c
HD
SYNTH
BoRSH3_a
HD
SYNTH
BoRSH3_b
HD
SYNTH
BrRSH3
HD
SYNTH
CsRSH3_a
HD
SYNTH
CsRSH3_b
HD
SYNTH
RsRSH3
HD
SYNTH

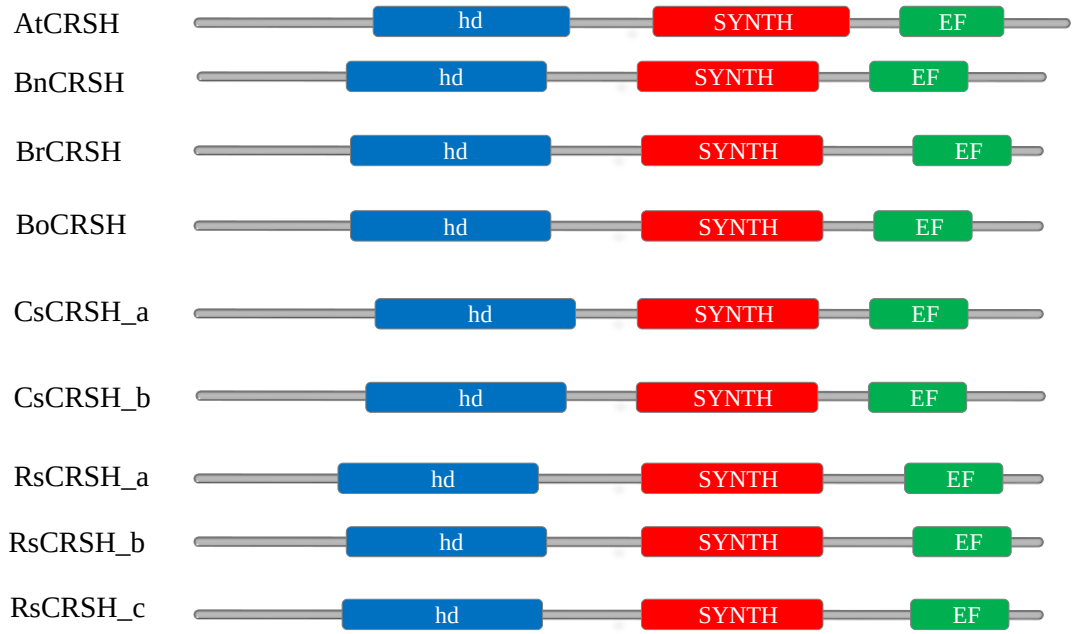

Supplementary Figure S2. Predicted primary structures of RSH1, RSH2/RSH3 and CRSH proteins from *A. thaliana*, *B. napus*, *B. olearacea*, *B. rapa*, *C. sativa* and *R. sativus*. HD (p)ppGpp hydrolase domain; SYNTH (p)ppGpp synthase domain; ACT aspartate kinase chorismate mutase TyrA domain; EF Ca^{2+}-binding domain; TGS: threonyl-tRNA synthetase, GTPase, SpoT domain.

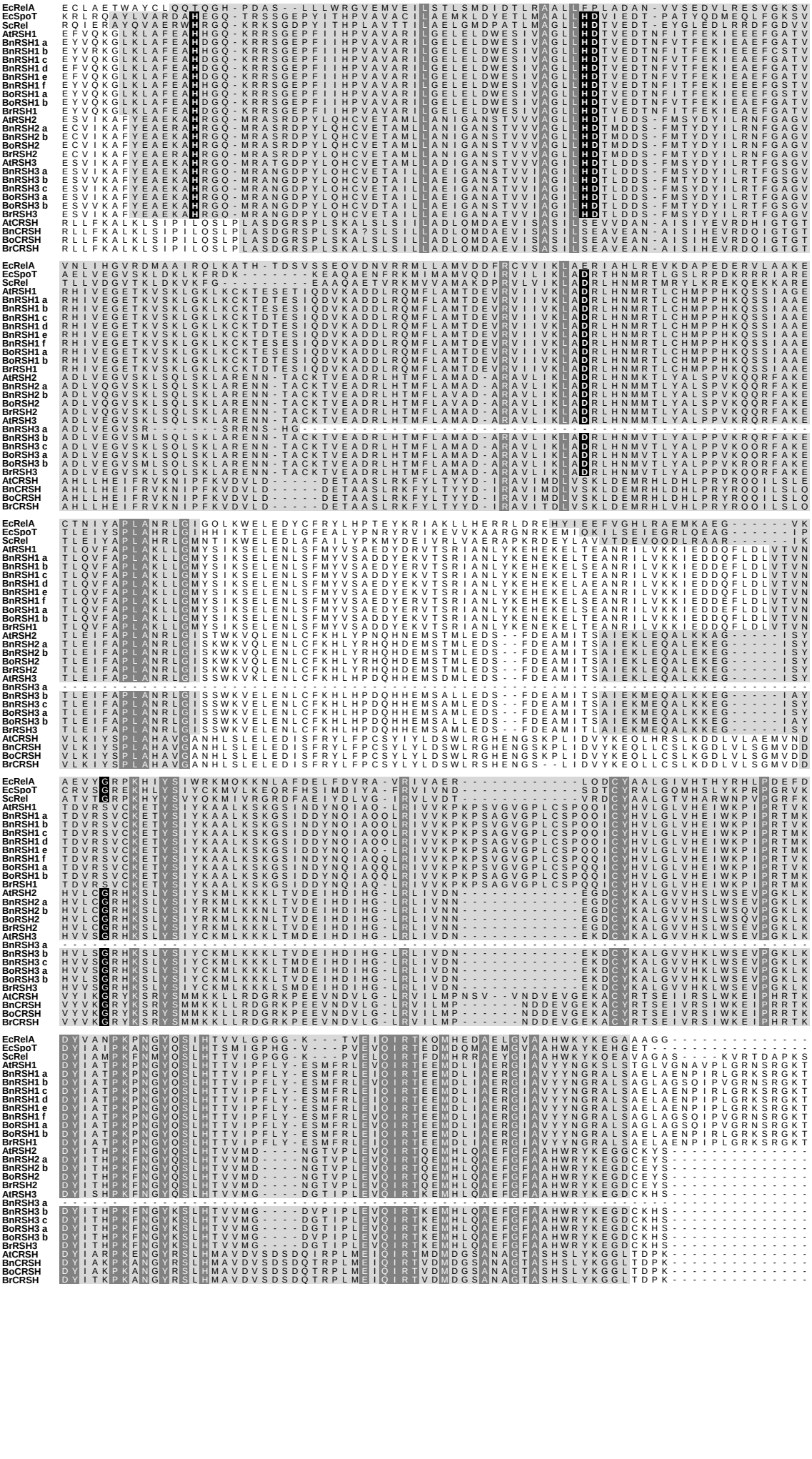
EcRelA
EcSpoT
ScRel
AtRSH1
BnRSH1 a
BnRSH1 b
BnRSH1 c
BnRSH1 d
BnRSH1 e
BnRSH1 f
BoRSH1 a
BoRSH1 b
BrRSH1
AtRSH2
BnRSH2 a
BnRSH2 b
BoRSH2
BrRSH2
AtRSH3
BnRSH3 a
BnRSH3 b
BnRSH3 c
BoRSH3 a
BoRSH3 b
BrRSH3
AtCRSH
BnCRSH
BoCRSH
BrCRSH

Supplementary Figure S3. Comparison of the deduced amino acid sequences of the (p)ppGpp hydrolase/synthetase domains between plants (*A. thaliana*, *B. napus*, *B. olearacea*, *B. rapa*, *C. sativa* and *R. sativus*) and bacterial RSHs. The first section highlighted in grey represent HD domain, the second section highlighted in grey represents SYNTH domain.

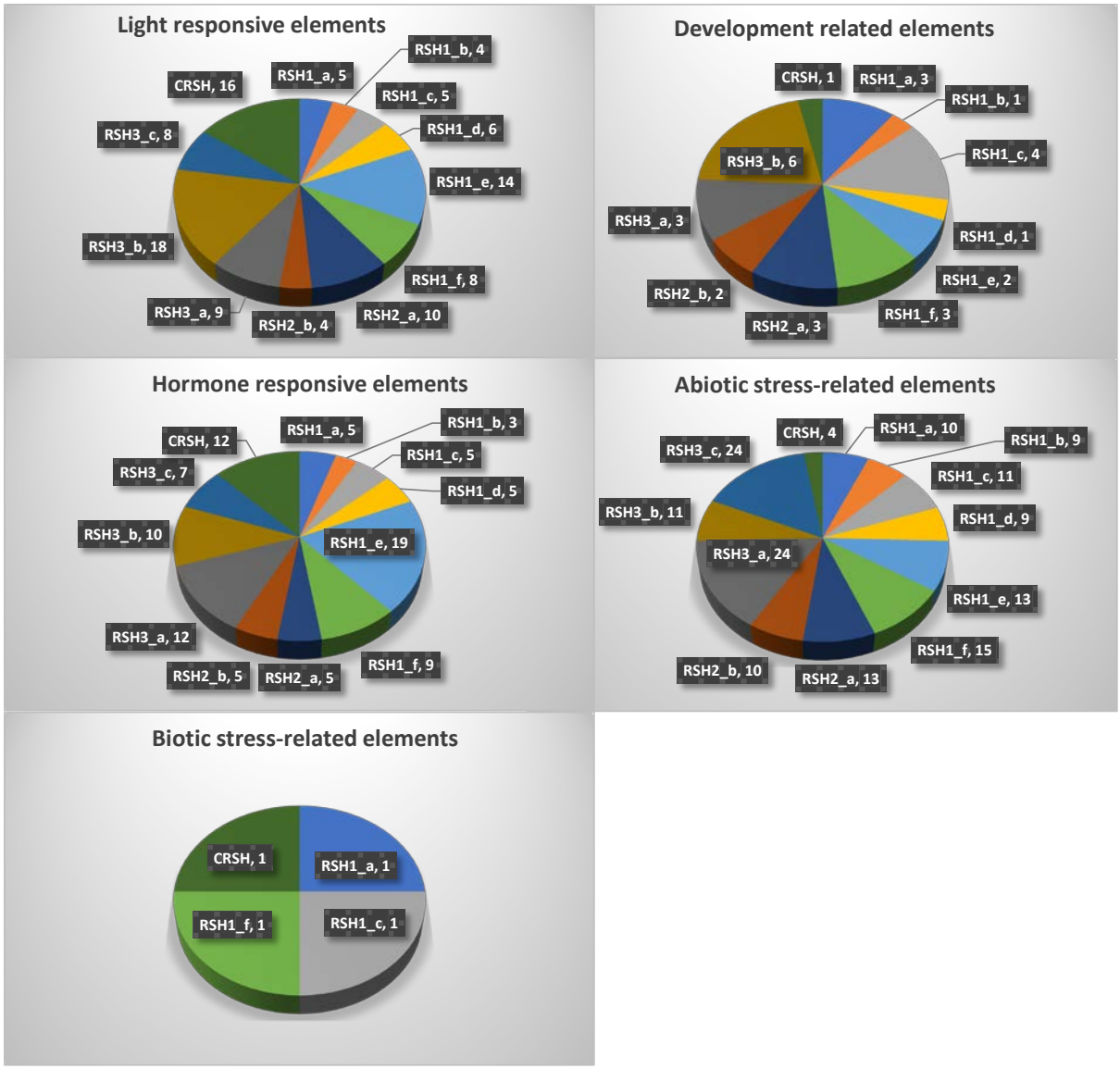

Supplementary Figure S4. The frequencies of cis-regulatory elements in promoter regions of *BnRSH* genes. Regulatory elements were grouped according to their functions. The exact number of particular types of regulatory element in each gene is given next to gene name.

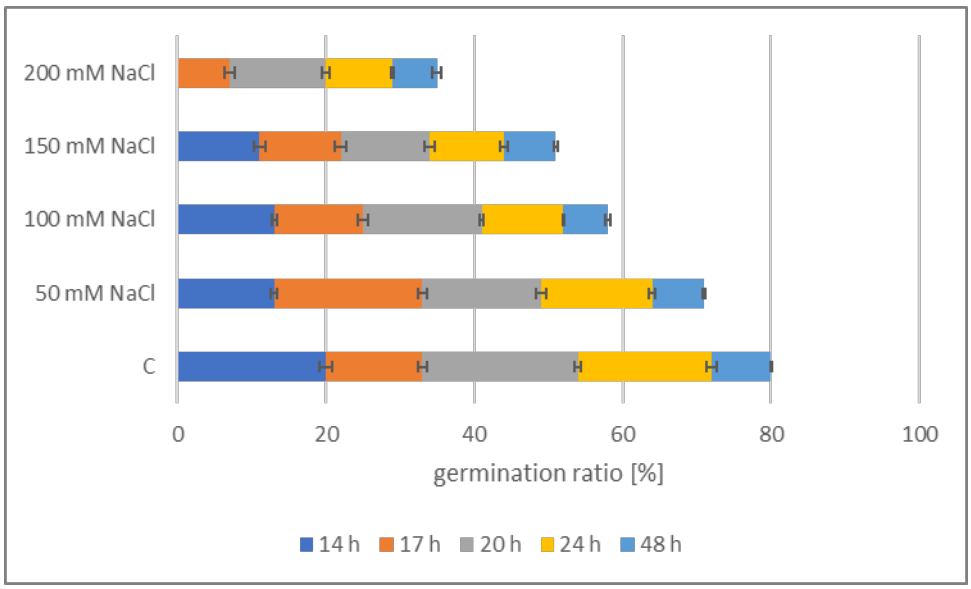

Supplementary Figure S5. The effect of NaCl on germination ratio of *B. napus* seeds. Seeds were germinated in the presence of NaCl (50, 100, 150, 200 mM) or in water (C) and the number of germinated seeds was checked after 14 h, 17 h, 20 h, 24 h and 48 h. The bars expressed the percent of germinated seeds to the total number of seeds ± SD. The experiment was repeated three times with 100 seeds per replicate per each treatment.

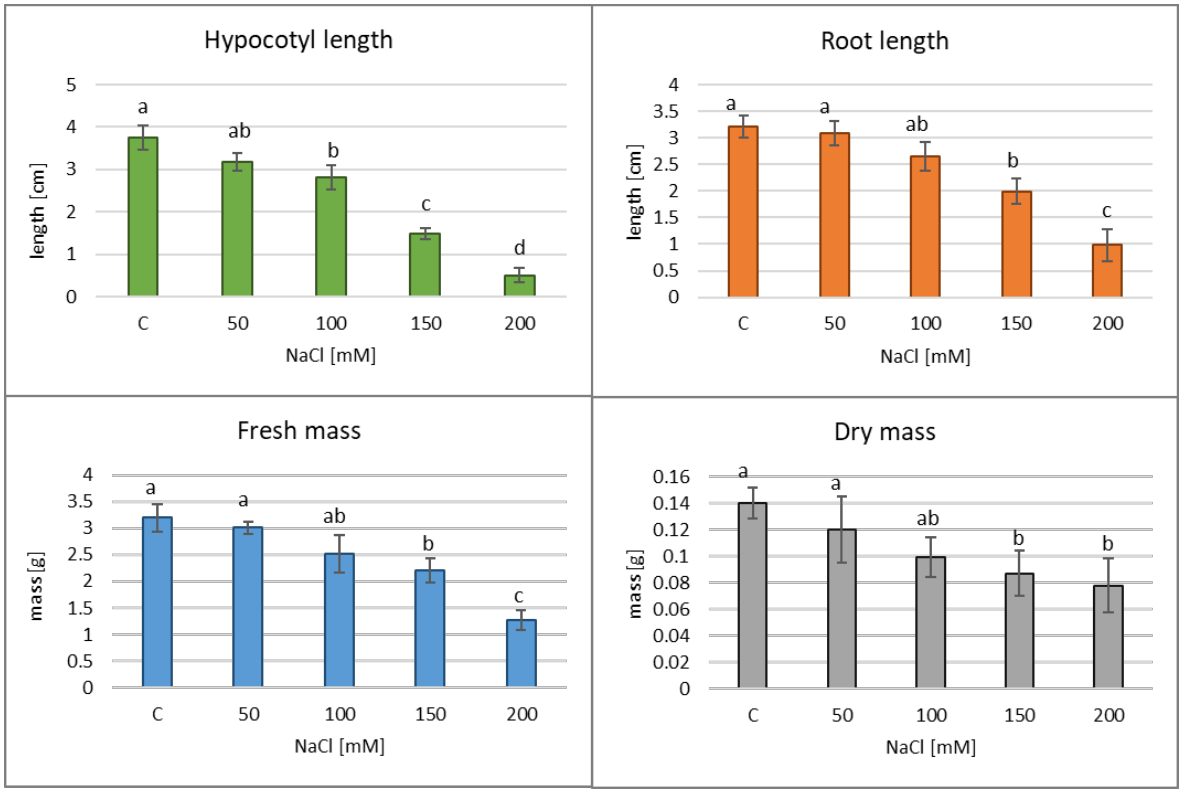

Supplementary Figure S6. The growth of 6-day-old *B. napus* seedlings in the presence of NaCl (50 mM, 100 mM, 150 mM and 200 mM) or in water (C). The hypocotyl length (B), root length (C), fresh mass (D) and dry mass (E) of 6-day-old seedlings was measured. The graphs show means from three independent biological replicates (50 seedlings for each replicate) ± SD. The statistical analysis was performed using one-way ANOVA followed by Tukey's honest significance test using R version 4.1.1. Different letters indicate statistically significant differences at p-value < 0.05.
